# The Relationships between Character Strengths and Subjective Wellbeing: Evidence from Greece under Lockdown during COVID-19 Pandemic

**DOI:** 10.3390/ijerph182010868

**Published:** 2021-10-15

**Authors:** Dimitra Vasileiou, Despina Moraitou, Vasileios Papaliagkas, Christos Pezirkianidis, Anastasios Stalikas, Georgia Papantoniou, Maria Sofologi

**Affiliations:** 1 Laboratory of Psychology, Section of Experimental and Cognitive Psychology, Faculty of Philosophy, School of Psychology, Aristotle University of Thessaloniki, 54124 Thessaloniki, Greece; demorait@psy.auth.gr; 2 Laboratory of Neurodegenerative Diseases, Center for Interdisciplinary Research and Innovation (CIRI—AUTH), Aristotle University of Thessaloniki, 57001 Thessaloniki, Greece; gpapanto@uoi.gr; 3 Department of Biomedical Sciences, Alexander Campus, International Hellenic University, P.O. Box 141, 57400 Thessaloniki, Greece; vpapal@auth.gr; 4 Lab of Positive Psychology, Department of Psychology, Panteion University of Social and Political Sciences, 17671 Athens, Greece; christospez@hotmail.com (C.P.); anstal@panteion.gr (A.S.); 5 Laboratory of Psychology, Department of Early Childhood Education, School of Education, University of Ioannina, 45110 Ioannina, Greece; m.sofologi@uoi.gr; 6 Institute of Humanities and Social Sciences, University Research Centre of Ioannina (URCI), 45100 Ioannina, Greece

**Keywords:** eudaimonic wellbeing, hedonic wellbeing, positive psychology, COVID-19, character strengths

## Abstract

COVID-19 was first identified in December 2019. As long as this type of coronavirus was new, the main way for governments to avoid the spread of the infection was enforced quarantine. Besides public health protection, quarantine can have a psychological impact on the residents, with main symptoms being angst, anxiety, depressive, and PTSD symptoms. As it has been found that character strengths can promote subjective wellbeing, the purpose of the study was to examine this relationship under the new situation of quarantine in the Greek population in adults who were in quarantine for at least two weeks. The total sample consisted of 354 participants who were aged 18–72-years-old. A total of 263 participants were women (74.3%), 91 were men (25.7%), and 94.6% of them were highly educated. The sample was a convenience sample. The tools used were PANAS, PERMA and finally VIA-114GR. The data analysis was completed using SPSS software version 26.0 (IBM Corp. Released 2019. IBM SPSS Statistics for Windows, Version 26.0. Armonk, NY, USA: IBM Corp) and EQS 6.1 (Multivariate Software Inc.: Encino, CA, USA, 2006). The results showed that love, curiosity, persistence, hope, and zest are strongly associated with subjective wellbeing, even in conditions such as quarantine, and can support specific aspects of it.

## 1. Introduction

In December 2019 in Wuhan, China, a new type of coronavirus was first identified, which leads to coronavirus disease 2019 (COVID-19), a disease caused by the severe acute respiratory syndrome “coronavirus 2” (SARS- CoV-2) [1]. The severity of the disease differs from mild to severe [2], and it seems to be highly contagious [3,4,5]. The main symptoms are cough, fever, and breathlessness; however, a lot of patients who are positive for the virus have no symptoms [3]. The WHO announced the COVID-19 outbreak as a pandemic on 11 March 2020. A pandemic is a situation where a rather infectious disease spreads worldwide in a short period of time [1]. 

As this type of coronavirus was new, there was neither an effective treatment nor a vaccine [3,6]. This led to the implementation of a series of prevention strategies by the governments of affected countries, including rules relating to hygiene, social distancing, and quarantine [3]. Quarantine (voluntary or enforced) is a state where people need to be isolated for a specific period of time in a specific place in order to prevent the spread of an infectious disease to other people. Although, being in quarantine may promote the health of people, it may also have a negative effect on their emotional and social health [7]. This negative aspect of quarantine has also been mentioned in other similar outbreaks, such as SARS (2003) and H1N1 (2009) [7].

Specifically, being in quarantine can cause people to feel angst concerning their futures, the possible implementation of new preventive measures, and the potential scarcity of essentials [7,8]. Moreover, it has been observed that people in quarantine are rather concerned about their own and their loved ones’ wellness [7,9,10], regardless of whether they are infected or not [11]. Augmented difficulty is also observed when populations are asked to stay at home, and there are constraints on their transportation, hobbies, and ability to meet with their loved ones [9,10], resulting in expressions of disappointment and boredom [7,10,12]. Another element that should be taken into consideration is the duration of the quarantine, as it seems to affect the severity of the psychological consequences [7,9,10,11].

The main psychological symptoms sighted under quarantine circumstances are anxiety/stress, depression, and post-traumatic stress disorder (PTSD) [7,9,12,13,14,15,16]. It seems that the augmented stress levels observed at the peak of the outbreak are retained even after the end of it, mostly because of the constant references to the outbreak by the media [8]. Some researchers support that these symptoms may be detected weeks, months, or even years after the end of the quarantine [7]. This leads us to wonder how important it is to include effective mitigation measures in quarantine planning procedures [7].

Until now, besides a history of chronic psychological disorders, there have been no recorded elements of a person’s characteristics that have been shown to effectively predict the physiological impact of quarantine on an individual [7,8,17]

It would be particularly important and innovative to find which characteristics make people capable of overcoming difficulties and not be significantly affected by difficult and stressful situations while maintaining high levels of wellbeing.

However, there are some people who can stay calm despite the chaos around them. Positive psychology seems to answer this question, that is, how people deal with adverse situations such as a pandemic, which leads to a preventive quarantine. Positive psychology focuses on what makes life worth living. It is not about recovering from anything problematic, but centers on every positive element that each person has in their life and aims to develop a person’s potential so that they are able to thrive. Its purpose is high levels of functioning through the development of each positive human characteristic [18]. Specifically, among the central concerns of positive psychology are people’s character strengths [19].

Character strengths are the positive traits that enable people to fulfill their potential [19,20]. It has been found that higher levels of character strengths are correlated with higher levels of life satisfaction, which leads to lower levels of psychological and social problems and higher levels of function in domains such as interpersonal relationships, work performance, physical health, etc. [18]. 

Character strengths are the main psychological components of virtues. Virtues are universal characteristics and may have a biological groundwork. They are selected through an evolutionary process, as they help people survive [21]. In the Greek population, a model of 5 virtues composed of 24 character strengths has been established. These virtues are the interpersonal virtue and the virtues of intellect, restraint, knowledge, and transcendence [22]. In particular, it has been found that kindness, love, honesty, fairness, and persistence are the five strengths that are the most common virtues in Greek people [22]. Table 1 shows the dimensions of every virtue, that is, which character strengths each virtue consists of. 

There seems to be a difference according to age, with adults who are 55–64 years old reporting higher levels of almost all of the restraint dimensions, while adults who are 18–24-years-old have the lowest score in terms of prudence and self-regulation. Moreover, participants aged 45- and 54-years-old demonstrate the highest levels of the transcendence virtue and the respective strengths of that virtue. Alongside age differences, gender differences also have been noted; for example, women reported higher levels of kindness and appreciation of beauty, while men reported higher level of creativity, humor, curiosity [22]. 

### 1.1. Literature Review

Complementarily, subjective wellbeing has also had a strong association with experiencing less negative emotions, such as those caused by quarantine, and in arousing positive emotions and feelings of flourishment [23]. Most researchers agree that there are two concepts of wellbeing, hedonic and eudaimonic [24]. Hedonic wellbeing is about pleasure, enjoyment, comfort, satisfaction, and ease and centers on what somebody wants in the present moment. Positive affect is dominant, and negative affect is represented at a low degree [25]. On the other hand, eudaimonic wellbeing is about meaning, value, personal growth, maturity, self-realization, excellence, quality, accomplishments, and engagement. It focuses equally on both the present and the future. Nevertheless, there are some common elements between the two concepts, such as life satisfaction and subjective value [25]. Most studies suggest that people need both aspects of wellbeing to flourish. They are not two opposite elements; in contrast, one complements the other, so it is necessary for both variables to be considered when subjective wellbeing and its outcomes are being studied [25]. Hence, in this study, we wanted to examine both eudaimonic wellbeing and hedonic wellbeing. 

Various theories have attempted to determine the main components of subjective wellbeing [26]. However, the most recent working multidimensional theory is Seligman’s, 2011 [20] PERMA theory. There are five components of subjective wellbeing: (a) positive emotions: experiencing positive emotions leads to wellbeing; (b) engagement: dedication and subsequent satisfaction after physical, cognitive, and emotional activity; (c) positive relationships: healthy and supportive relationships with family, friends, and other important people; (d) meaning in life: coming across a way to make life worth living; (e) accomplishments, the afterglow of accomplishments as a mainspring for action [26].

There is a positive correlation between character strengths and subjective wellbeing [27,28]. In particular, it has been found that in the Greek population, all character strengths except for modesty, love of learning, appreciation of beauty, and self-regulation have a positive correlation to all five PERMA factors and overall wellbeing. The strengths of love, hope, curiosity, and zest are more correlated to all of the PERMA’s dimensions [22]. On account of this, there have been many studies that have created interventions that aimed to develop or enhance character strengths in order to increase wellbeing [29,30].

Character strengths can be defined as positive traits that are reflected in thoughts, feelings, and behavior and have been linked with subjective wellbeing (SWB) [31]. SWB is reported as the different ways in which individuals appraise their quality of life and is about an interaction between cognitive and emotional elements [31]. The main components of SWB are life satisfaction, higher levels of positive affect, and lower levels of negative affect in everyday experiences [32,33]. In the same vein, character strengths are correlated to life satisfaction, positive emotions, and orientation to happiness [22]. Accordingly, there is a correlation between character strengths and SWB in terms of life satisfaction as a cognitive aspect of wellbeing [19]. The more strongly the strength is valued, the more life satisfaction is reported [19]. Some researchers have indicated that some character strengths are more related with SWB. Moreover, character strengths play a significant and indirect role in depression through the mediation of dysfunctional attitudes, negative affect, and happiness [30]. 

It has been noted that character strengths have a negative effect on dysfunctional attitudes and a positive effect on happiness [30]. Additionally, negative affect is the only element that has been found to have a direct impact on the depressive symptoms [30] that pandemics and quarantines can cause [12,13,14,15,16]. People with high levels of character strength have high levels of happiness too [30]. Happiness has an immediate effect on negative affect, and as a result, it has an indirect effect on depressive symptoms, either by increasing people’s resilience against the noxious effects of early adversities and stressors or by reducing the effects of negative emotions on depression [30,33]. In contrast, people with low levels of character strength cultivate maladaptive attitudes toward oneself and as a result, produce dysfunctional attitudes to average out these not suitable statements for themselves. Dysfunctional attitudes have an indirect effect on depression by increasing someone’s negative affect. It seems that character strength can reduce depressive symptoms without a significant inverse effect. However, positive and negative affect are two distinguishable elements. Hence, by increasing positive affect, negative affect does not necessarily decrease [33]. According to positive psychopathology, psychosocial factors can attenuate everyone’s inherent ability for growth, fulfillment, and wellbeing. Furthermore, pre-treatment character strengths are related to post-treatment recovery from depression [30].

Positive psychology and character strengths help people to develop their potential, to have the maximum human experience, and to cope with any difficulty. From the other hand, mindfulness helps people to recognize the truth of the present moment. The combination of the two (character strengths and mindfulness) gives people the strength to overcome their weaknesses and thus to provide opportunities for their uncovered skills to flourish. In the same line, at present, the extent to which humans emphasize and use their appropriate character strengths depends on the context. This process, which is based on the function of attention and character strengths, helps people to seize every opportunity that comes their way. In fact, the term “strength spotting” refers to the ability of people to recognize their own behaviors and those of others and to define and name them, and this procedure activates the awareness of these strengths and their potential use. [34]. It has been found that mindfulness-based strength practice can lead to increased levels of wellbeing and physical health [35]. As such, it is very important to determine the character strengths that help people cope with difficult and unexpected conditions such as quarantines in order to use them with purpose. 

In this context, the purpose of this study was to analyze the relationship between character strengths and both hedonic and eudaimonic SWB in a quarantine context. We wanted to examine which character strengths are more related to SWB and the differences in this relationship according to age and gender. Finally, we would like to look into the moderating role of continuing to work from home and living with loved ones during quarantine.

### 1.2. The Present Study

The purpose of the study was to examine which character strengths potentially contribute to eudaimonic and hedonic wellbeing in people who have been quarantined for at least two weeks due to the coronavirus pandemic. In particular, we wanted to examine whether character strengths contribute to high levels of subjective wellbeing in the Greek population in the conditions of forced quarantine despite the negative psychological effects that it may cause. As positive psychology and, more specifically, character strengths seem to positively correlate to SWB in conditions of normality, we wanted to study whether the same is true in this particular condition of a pandemic and the subsequent quarantine. We also wanted study the moderating role of individual demographic factors (age, gender, working from home, living alone) in terms of this relationship.

**Figure 1:**
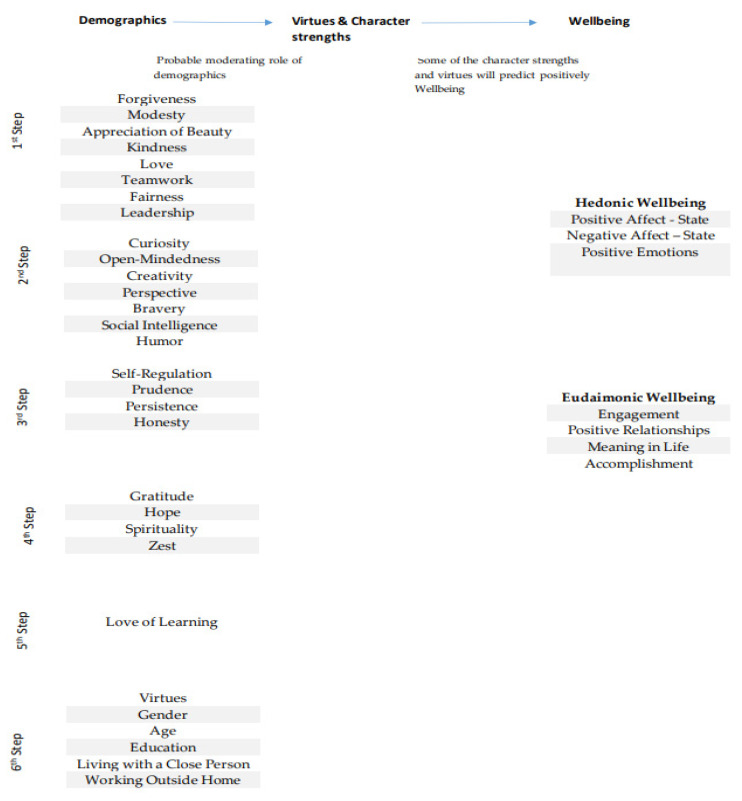
Research Hypothesis.

It was expected that at least some character strengths would correlate positively SWB, similarly to what has been reported in the literature under normal conditions (Hypothesis 1) [22]. Additionally, there was a research question regarding the extent to which this relationship would be positively or negatively moderated by demographic factors, i.e., gender, age, education, living with a close person, not working from home (see Figure 1).

## 2. Materials and Methods

### 2.1. Participants

The study sample was Greek adults (N = 354) who were 18 to 72 years of age. The mean age of the total sample was 32.08 years (*SD* = 10.94). There were 263 women (74.3%) and 91 men (25.7%). Thus, female gender was overrepresented in the sample. Regarding the educational level, the majority of the participants had 13 or more years of education (n = 335, 94.6%). Hence, almost all of the participants were of a high educational level. Regarding the duration of their quarantine, when the study took place, the average was 2.76 weeks (*SD* = 1.05). Additionally, 285 (80.5%) of the participants stated that they lived with another person, while 69 (19.5%) stated that they lived alone. When they asked if they still worked outside their home during quarantine, 71 people answered in the affirmative (20.1%).

### 2.2. Measures 

Demographics: Participants were asked to report demographic information including gender, age, education, weeks of quarantine, if they were living alone, and still working outside of their home during quarantine. 

The Positive and Negative Affect Schedule (PANAS) (Watson, Clark & Tellegan, University of Minnesota and Southern Methodist University, 1988) [36] (Greek adaptation: Efklides & Moraitou, Greece, Thessaloniki, 2009) [37]. The questionnaire consists of 20 items—10 representing negative affect and 10 representing positive affect [36,37]. The scale measures positive emotions, such as proud and active, and negative emotions, such as guiltiness and fear. A study in the Greek population confirmed the existence of two factors in the structure of the scale, the positive and the negative affect, each of which includes ten emotions [37]. Responses are given to a 5-point Likert-type scale from “1-very few times or not at all” to “5 too many times”. Participants answered to what extent they felt what was described by each item over the last two weeks. As such, state affects were measured via PANAS administration. The developers reported high levels of internal reliability in all of the measurements that were taken (as state or trait affect), with Cronbach’s alpha ranging from 0.86 to 0.90 for the positive affect subscale and from 0.84 to 0.87 for the negative affect subscale [36]). In the Greek adaptation, Cronbach’s alpha was α = 0.84 for positive affect and α = 0.82 for negative affect. In the present study, Cronbach’s α was 0.088 for the state negative affect and 0.85 for the state positive affect. 

PERMA Profiler (Butler & Kern, Melbourne, Australia, 2016) [38], (Greek version: Pezirkianidis et al., Greece, Athens, 2019) [26]. This multidimensional questionnaire consists of 23 items that measure the five pillars of subjective (hedonic and mainly eudaimonic) wellbeing [38]. According to Seligman’s theory, 2011 [20] these pillars are positive emotions, engagement, positive relationships, meaning in life, and accomplishments. It also includes eight additional items: a single item for satisfaction with life and for loneliness and three items for negative emotions and for physical health. There is also an overall score for all of the items of the PERMA and the single item for satisfaction with life. Responses are given to an 11-point Likert-type scale anchored by “0-Never/Not at all/Terrible” to “10-Always/Completely/Excellent” to answer each item. The Greek validation of the PERMA profiler confirmed the five-factor structure of the instrument and revealed acceptable internal consistency as well as adequate convergent and discriminant validity. More specifically, for every one of the five pillars of wellbeing, Cronbach’s α was α = 0.83 for positive emotions; α = 0.56 for engagement; α = 0.74 for positive relationships; α = 0.78 for meaning in life; and α = 0.72 for accomplishment. Hence, the five PERMA subscales are characterized by adequate levels of reliability, apart from the engagement subscale which shows a low level of internal consistency [26]. Same results are showed in the present study. Cronbach’s α was 0.87 for positive emotions, 0.58 for engagement, 0.76 for positive relationships, 0.81 for meaning in life, and 0.76 for accomplishment. The internal consistency of the total PERMA in the present study was found to be α = 0.91.

Values In Action—114GR (VIA-120: Peterson & Seligman, New York: Oxford University Press and Washington, DC: American Psychological Association, 2004) [21], (Greek translation: Dimitriadou & Stalikas; Pedio: Greece, Athens, 2012) [39]. The VIA-114GR (Pezirkianidis et al., 2020, Athens, Greece) is the Greek version of the VIA-120 and was conceptualized in the framework of Greek culture [22]. VIA-114GR contains 114 items and measures five virtues and twenty-four character strengths according to the classification of Peterson and Seligman, 2004 [21]. Responses are given on a 5-point Likert-type scale labeled “0-Very much like me to 4-Very much unlike me”, and the participant has to report the extent to which each item describes them. The VIA-114GR demonstrates good internal reliability, convergent validity regarding wellbeing indices, and discriminant validity regarding negative experiences. The internal consistency of the total VIA-114GR was found to be α=0.96 [22]. The internal consistency of the 24 character strengths was tested, and the results showed that the adequate reliability for almost all of the strengths ranged from α = 0.70 to α = 0.82 for the 20 strengths. However, four strengths, open-mindedness (α = 0.62), fairness (α = 0.66), modesty (α = 0.60), and self-regulation (α = 0.65) showed marginal reliability coefficient values. Cronbach’s α for the five virtues was 0.92 for the interpersonal virtue, 0.91 for the virtue of intellect, 0.86 for the virtue of restraint, 0.89 for the virtue of transcendence, and 0.81 for the virtue of knowledge [22]. Similar results were found in the present study. More specifically, 18 out of 24 character strengths showed adequate to very good reliability, which ranged from α = 0.72 to α = 0.87. However, honesty (α = 0.68), social intelligence (α = 0.66), forgiveness (α = 0.60), fairness (α = 0.62), modesty (α = 0.58), and open-mindedness (α = 0.59) showed marginal to low reliability coefficient values. Cronbach’s α for the five virtues was 0.82 for the interpersonal virtue, 0.81 for the virtue of intellect, 0.71 for the virtue of restraint, 0.86 for the virtue of transcendence, and 0.77 for the virtue of knowledge. The internal consistency of the total VIA-114GR in the present research was found to be α = 0.90.

### 2.3. Procedure

We initially constructed an electronic questionnaire that included the information and the consent form, and all of the the above measurements. This questionnaire was accessible to any device with internet connection. Accordingly, the questionnaire was posted on social media (e.g., Facebook) to university and cultural groups. The research was conducted online because it was required to meet quarantine conditions and took place during quarantine, so participation was not possible in any other way. Participation in the study was anonymous and was voluntary, and theconvenience sampling method was used. Τhe only exclusion criteria were age (only adults over 18 years of age were permitted to participate) and quarantine weeks (2 weeks and over). The study was conducted during the quarantine period from March to May 2020. This study was approved by the Scientific Research Ethics Committee, Aristotle University of Thessaloniki, School of Psychology (037/04-04-2020).

### 2.4. Ethical Standards

The authors assert that all procedures contributing to this work comply with the ethical standards of the relevant national and institutional committees on human experimentation and with the Helsinki Declaration of 1975, as revised in 2008. All participants participated in the study voluntarily. They were informed about the procedure and the aim of the study, and subsequently, they provided their written consent for participation. The study was approved by the Scientific Ethics Committee of the School of Psychology of the Aristotle University of Thessaloniki and was fully in line with the European Union Regulation on sensitive personal data (28 May 2018).

## 3. Results

The statistical data analysis package for social sciences SPSS v.260 (IBM Corp. Released 2019. IBM SPSS Statistics for Windows, Version 26.0. Armonk, NY: IBM Corp) [40] and the statistical program for structural equation modeling EQS 6.1 (Multivariate Software Inc.: Encino, CA, USA, 2006) [41] were used for the analysis. Twenty-nine variables were created as the sum of the items that were found to constitute the respective psychological quality in previous structural–factorial analyses (see the Section 2.2) for character strengths and virtues (twenty-four character strengths and five virtues) and seven were created for the subjective wellbeing components (five PERMA factors, state positive affect, and state negative affect). For these variables, descriptive statistics (average, standard deviation, minimum, and maximum values) were calculated. Subsequently, a series of path analyses was performed to examine whether the character strength variables could affect the components of subjective wellbeing and secondly, whether individual demographic characteristics affect both character strengths and SWB. Due to the sample size constrictions, we decided to examine whether each group of character strengths constituting a specific virtue could predict the level of every SWB component. Hence, we finally confirmed five path models, one for each of the strengths comprising each virtue. Another model in which we entered the five virtues as predictors of wellbeing and demographic factors as predictors of both virtues and wellbeing components was finally confirmed. The five virtue variables were created by summing the scores of the strengths that constitute each virtue.

Before presenting the confirmed path models, it is important to mention the model fit indicators. To support the goodness of fit of a path model to the data, the level of statistical significance for the goodness of fit index χ^2^ should be *p* > 0.05. In addition, the root mean square error of approximation (RMSEA) value must be less than 0.05 to approximately indicate the good fit of the model to the data, while RMSEA index values between 0.06 and 0.08 indicate a reasonable and therefore acceptable error of approximate fit. Regarding the comparative fit Index (CFI), which evaluates the fit of the proposed model in relation to a limited, basic model, values greater than 0.90 indicate a sufficient fit of the model to the data [42].

**Figure 2:**
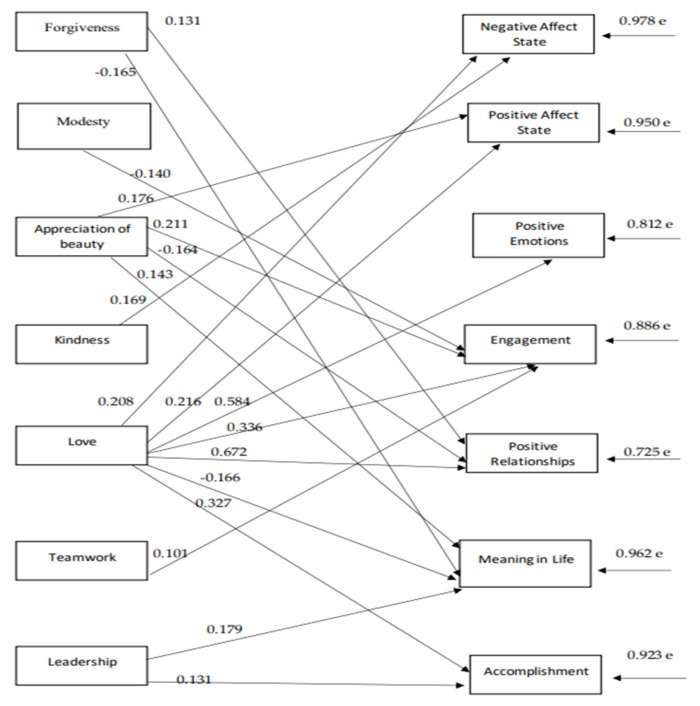
The relationships between interpersonal virtue dimensions and subjective wellbeing components.

The path model indices, which were confirmed for the strengths of the 1st virtue, namely the interpersonal virtue (Figure 2), indicate a satisfactory model fit to the data, χ^2^(47) = 72.95, *p* = 0.009, CFI = 0.98, SRMR = 0.04, RMSEA = 0.04 (90% CI: 0.02–0.06). According to the 1st model, “love” is the character strength that predicts all of the components of both hedonic and eudaimonic wellbeing. “Love” only negatively predicts “meaning in life”. The highest positive relationship for love is with “positive relationships”, and the lowest relationship is with “state negative affect”. “Appreciation of beauty” positively predicts “state positive affect”, “engagement”, and “meaning in life” and negatively affects “positive relationships”. “Forgiveness”, positively predicts “positive relationships” and negatively affects “meaning in life”. “Modesty”, negatively predicts “engagement”, while “kindness” positively predicts “state negative affect”. “Teamwork” positively predicts “engagement”. “Leadership” positively predicts both “meaning in life” and “accomplishment” (see Figure 2). There are also reasonable correlations between the components of wellbeing with each other and between the character strengths with each other (see Table 2). 

**Figure 3:**
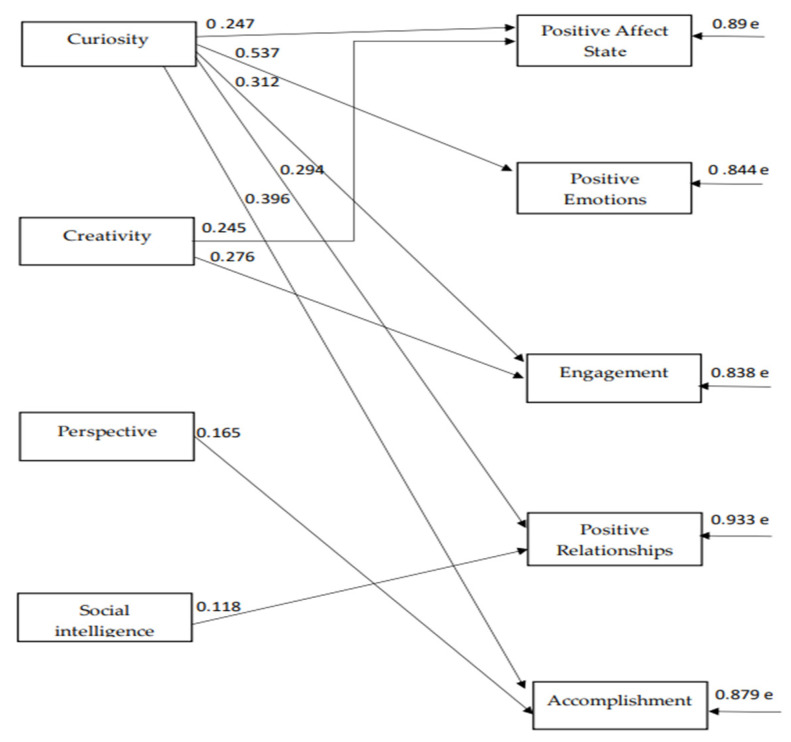
The relationships between virtue of intellect dimensions and subjective wellbeing component.

The path model indices, which were confirmed for the strengths of the 2nd virtue, namely the virtue of intellect (Figure 2), indicate a satisfactory model fit to the data, χ^2^(48) = 79.56, *p* = 0.002, CFI = 0.98, SRMR = 0.04, RMSEA = 0.04 (90% CI: 0.02–0.06). According to the 2nd model, “curiosity” is the character strength that positively predicts almost all of the components of both hedonic and eudaimonic wellbeing. “Creativity” positively predicts ”state positive affect” and “engagement”, meanwhile “perspective” positively predicts ”accomplishment”, and “social intelligence” positively predicts “positive relationships” (see Figure 3). There are reasonable correlations between the components of wellbeing and between character strengths with each other (see Table 3).

**Figure 4:**
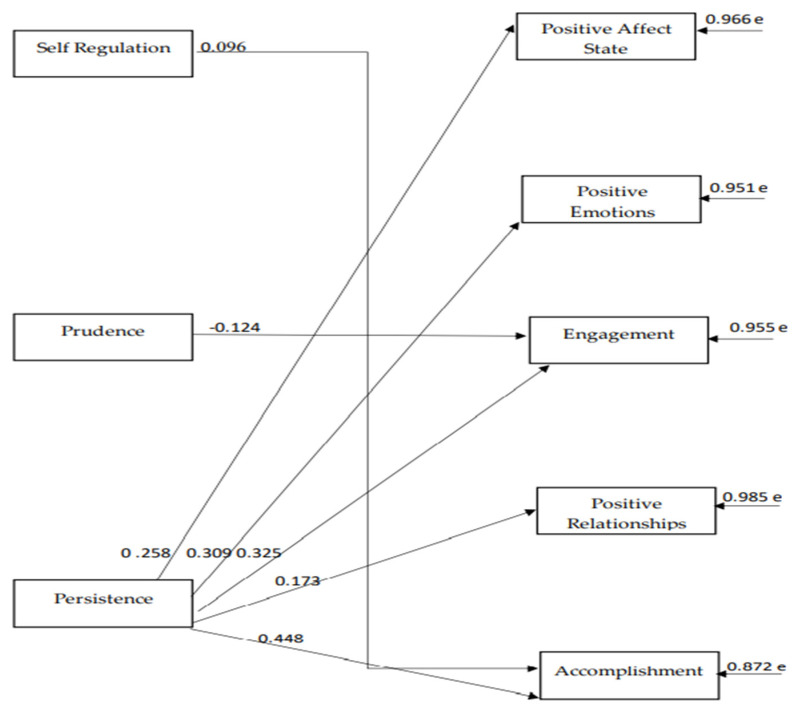
The relationships between virtue of restraint dimensions and subjective wellbeing components.

The path model indices, which were confirmed for the strengths of the 3rd virtue, namely the virtue of restraint (Figure 4), indicate a particularly satisfactory model fit to the data, χ^2^(26) = 34.14, *p* = 0.131, CFI = 0.99, SRMR = 0.04, RMSEA = 0.03 (90% CI: 0.00–0.06). According to the 3rd model, “persistence” is the character strength that positively predicts almost all of the components of both hedonic and eudaimonic wellbeing. “Prudence” negatively predicts “engagement”, and “self-regulation” positively predicts “accomplishment” (see Figure 4). There are reasonable correlations between the components of wellbeing and between the different character strengths with each other (see Table 4).

**Figure 5:**
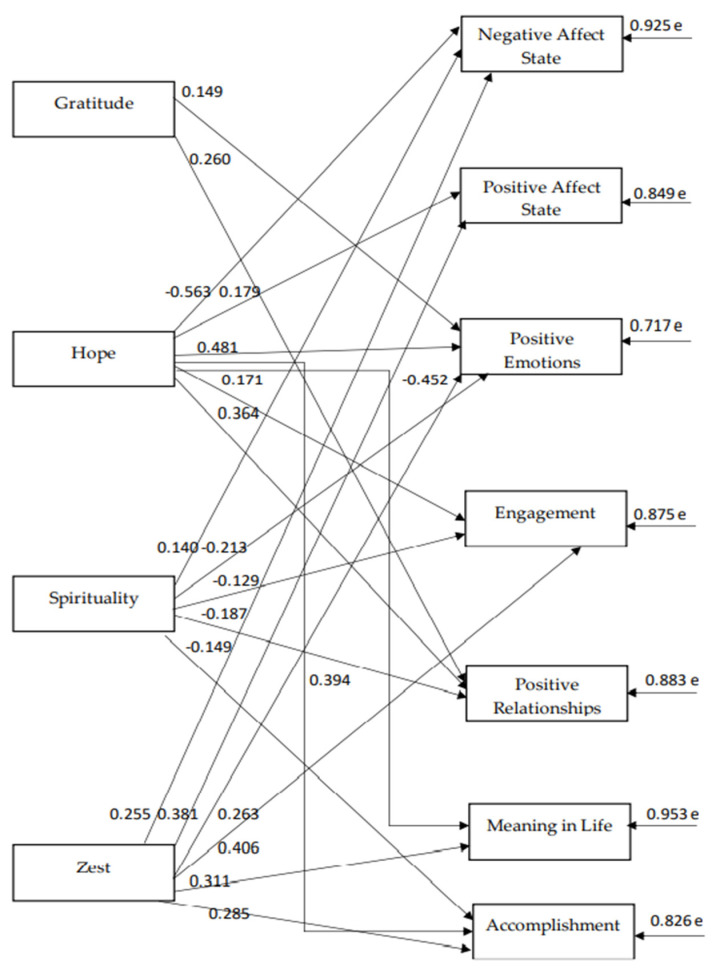
The relationships between transcendent virtue dimensions and subjective wellbeing components.

The path model indices, which were confirmed for the strengths of the 4th virtue, namely the transcendent virtue (Figure 4), indicate a particularly satisfactory model fit to the data, χ^2^(16) = 15.95, *p* = 0.456, CFI = 1.00, SRMR = 0.021, RMSEA = 0.00 (90% CI: 0.00–0.05). According to the 4th model, “hope”, “spirituality”, and “zest”, predict almost all of the components of subjective wellbeing. Namely, “hope” negatively predicts “state negative affect”, and “spirituality” negatively predicts “positive emotions”; “engagement”, “positive relationships”, and “accomplishments”. “Zest” has the highest positive relationship with “engagement” and the lowest with “state negative affect”. In addition, “gratitude” positively predicts “positive emotions” and “positive relationships” (see Figure 5). There are reasonable correlations between the components of wellbeing and between the different character strengths with each other (see Table 5).

**Figure 6:**
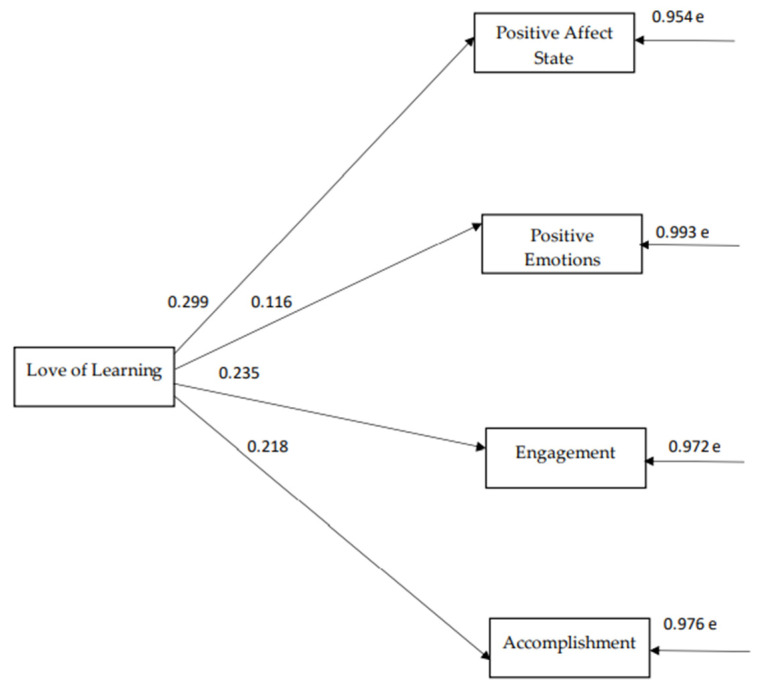
The relationships between virtue of knowledge dimensions and subjective wellbeing components.

The path model indices, which were confirmed for the strength of the 5th virtue, namely the virtue of knowledge (Figure 5), indicate a particularly satisfactory model fit to the data, χ^2^(7) = 10.6, *p* = 0.156, CFI = 0.99, SRMR = 0.03, RMSEA = 0.03 (90% CI: 0.00–0.08). According to the 5th model, “love of learning” positively predicts ”state positive affect”, “positive emotions”, “engagement”, and “accomplishment” (see Figure 6). There are reasonable correlations between the components of subjective wellbeing with each other (see Table 6).

**Figure 7:**
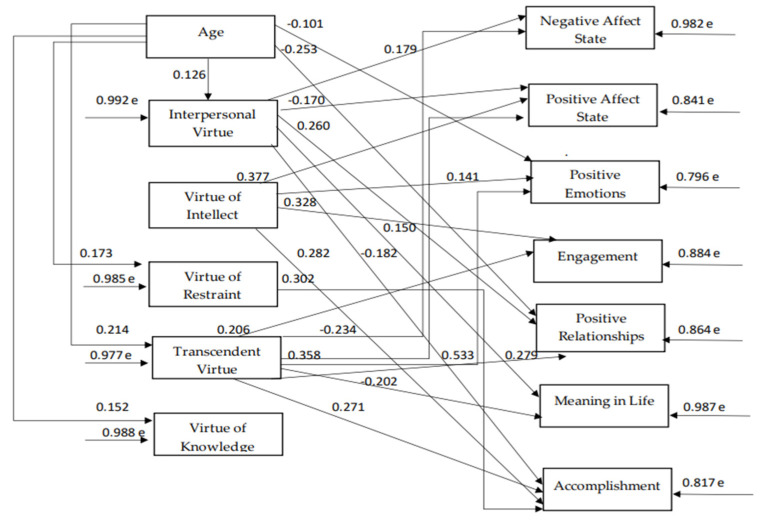
The relationships between demographics, virtues, and subjective wellbeing components.

Additionally, as mentioned above, there was a research question regarding the extent to which these relationships would be positively or negatively moderated by demographic factors, i.e., gender, age, education, living with a close person, work outside of the home. Due to sample size restrictions, these relationships were examined with virtue variables instead of with human strength variables. The path model indices, which were confirmed for the five virtues, demographics, and wellbeing components (Figure 7) indicate a satisfactory model fit to the data, χ^2^(31) = 44.6, *p* = 0.053, CFI = 0.99, SRMR = 0.04, RMSEA = 0.03 (90% CI: 0.00–0.06). According to the 6th model, “age” positively predicts the following virtues: “interpersonal virtue”, “virtue of restraint”, “transcendent virtue”, and “virtue of knowledge”. On the other hand, “age” correlates negatively with some wellbeing components, namely “positive emotions” and “positive relationships”, but “age” potentially has a positive relationship with wellbeing indirectly through these virtues that they are correlated with, as described below. Almost all virtues, except the “virtue of knowledge”, have both positive and negative relationships with wellbeing components. Specifically, the “virtue of transcendence” predicts all of the components of wellbeing positively, with the exception of “state positive affect” and “meaning in life”: these correlations are negative. “Interpersonal virtue” positively predicts “state negative affect”, “positive relationships”, and “meaning in life” and also has a negative correlation with “state positive affect” and “accomplishment”. “Virtue of intellect” positively predicts “state positive affect”, “positive emotions”, “engagement”, and “accomplishment”. “Virtue of restraint” only positively predicts “accomplishment”. There are reasonable correlations between wellbeing components with each other and between virtues with each other (see Table 7).

## 4. Discussion

The purpose of this study was to investigate the relationships between character strengths and both hedonic and eudaimonic wellbeing in the Greek population during pandemic due to COVID-19 in the middle of quarantine. More specifically, the study investigated whether character strengths can predict SWB and which ones have the highest role in enhancing SWB levels. The findings showed that the character strengths that are higher and mostly predict both hedonic and eudaimonic wellbeing positively are love, curiosity, persistence, hope, and zest. These findings are consistent with the extant literature, according to which love, hope, zest, and curiosity have the strongest effect on wellbeing [27,31,43] in general. However, fairness, open-mindedness, bravery, humor, and honesty were not found to have any significant relationship with any component of wellbeing, while spirituality was only found to negatively predict the components of wellbeing. Finally, age was found to have a moderating role in the aforementioned relationship.

### 4.1. The Relationships between Human Strengths and Affect as Positive Emotions, State Positive Affect and State Negative Affect (Hedonic Wellbeing)

Under the specific situation of quarantine, positive emotions, as a specific component of wellbeing, were positively predicted by love, curiosity, persistence, gratitude, hope, zest, and love of learning. There was also a negative correlation with spirituality.

In relation to state positive affect, as measured by the PANAS, it was positively predicted by appreciation οf beauty, love, curiosity, creativity, persistence, zest and love of learning. As for state negative affect, there were both positive and negative correlations. It was positively predicted by spirituality, zest, kindness, and love and was negatively predicted by hope. In the literature, all character strengths are usually positively or zero correlated to other wellbeing components (eudaimonic aspect) and zero or are negatively correlated to negative affect [22,27].

Broaden and build theory [44] describes the form and function of positive emotions, such as interest and love. First, these positive emotions expand a person’s momentary thought–action repertoire; for example, interest triggers the desire to explore, and love triggers close relationships (and the rest of the character strengths trigger behaviors as described below). Second, by expanding a person’s momentary thought–action repertoire, positive emotions promote the discovery of new and creative actions, ideas, and social bonds that lead to the construction of personal resources, physical, spiritual, social, and psychological resources. It is important that these resources act as reserves that can be used later to improve the chances of successful treatment and survival. This reaction to positive emotions is completely different from the reaction to negative emotions, which is a very limited reaction, and usually takes the form of fight or flight.

However, the pandemic and quarantine were very new and unknown situations [7,13], so people were not able to use their already-made personal resources for all of their character strengths due to these new circumstances, and they also had not developed broadened behaviors for every positive emotion that they had. This may be the reason why some character strengths positively predicted state negative affect, which is in contrast with the literature [22,27].

### 4.2. The Relationships between Character Strengths and the Engagement Component of Wellbeing

Under the specific conditions of quarantine, engagement as specific component of wellbeing was found to be the most positively predicted by zest as well as by hope, persistence, teamwork, creativity, curiosity, appreciation of beauty, love, and love of learning. Engagement is more of a state of mind rather than experiences and is about positivity and fulfillment. Additionally, engagement is about the effort that people make for a common good or achievement. Hence, engagement motivates people to work emotionally, behaviorally, and cognitively in order to achieve or create something [26]. 

Zest gives people the strength to work even under harsh conditions [26], such as quarantine, where people had to adapt to a new reality [45]. Hope positively predicted engagement, as it is a strength that gives people motivation for the future [46] because hope makes people more able to cope with the loss of their loved ones and more generally, to cope with disappointment and difficulties [47]. Persistence enhances engagement through the effort it applies to overcoming cognitive challenges [48] and to finishing every activity that one starts [20], which is more difficult in quarantine due to the various restrictions [45]. Teamwork positively predicted engagement because it promotes working together in harmony in order to achieve a common goal [20,26]. This strength is very important through quarantine, as people were confronted with new ways of working, studying, and communicating [45]. Creativity positively predicted engagement by considering new and productive ways to do things and curiosity by making ongoing experiences more interesting [20]. Especially, in a new, unexpected, and crucial period such as pandemics and quarantine, strengths such as creativity and curiosity were found to be extremely useful for people to cope with their new everyday life because they had to come up new ways of living, working, and entertaining themselves [46]. Appreciation of beauty is correlated with engagement by noticing skilled performance in all domains of life [20], as engagement is correlated to fulfillment, a strength such as appreciation of beauty could facilitate a much better adjustment to the requests of the environment. During this lockdown period, the population was required to learn different ways of working, examining, relaxing, and being at the side their nearest and dearest among other day by day propensities, allowing them to adapt better and resulting in higher wellbeing levels [46]. Commitment is a crucial component of love, as it promotes familiarity and similarity, which can increase engagement among people, a very important element of people relationships because it is in this way that relationships are more unfading over the course of many years [49], and during quarantine, relationships were tested [45]. Love of learning is about mastering abilities, which is the key component of engagement [20,27], and for a lot of people, quarantine was a period that was exploited to gain new knowledge and skills as well as to set new goals [45].

However, there were also found negative relationships with modesty, prudence, and spirituality. According to Wagner et al., 2019 [27] modesty and prudence are the character strengths that have not been related positively to wellbeing; in contrast, a small negative relationship has been noted with some of these with some components of wellbeing. Modesty was found to be one of the bottom strengths in a Greek sample too [22]. It has been suggested that these two character strengths do not help people to have high levels of wellbeing but that they do help to avoid negative experiences and also contribute to the feelings that people to help others, contributing to high levels of wellbeing [27]. As such, we did not expect to find a high positive correlation between these character strengths and some wellbeing components.

Based on the literature, spirituality can promote mental health, mainly through the reduction of stress levels in the body, through the power of faith. It can also lead to increased levels of altruism, happiness, and life satisfaction. It is suggested that by increasing levels of spirituality, levels of wellbeing also increase [50,51]. However, in a Greek sample, spirituality was one of the bottom strengths [22], which may partially explain the findings of the present study. Particularly, spirituality negatively predicted positive emotions, engagement, positive relationships, accomplishment, meaning in life and only positively predicted state negative affect. 

Spirituality brings believers closer to their faith. However, in order for people to have high levels of spirituality, some processes are required, such as expressions of admiration, devotion, exchange of beliefs, and worship. As such, it seems that religion is a very important component of spirituality [52]. Although, it has not been studied in its entirety, it seems that spiritual health contributes to the prevention and promotion of mental health [52].

In Greece, the majority of believers are Orthodox Christians, whose way of life, which offers them high levels of spiritual health, includes faith to their religion and contact with God through the sign of the Cross and through prayer. Additionally, it includes physical and spiritual fasting, the Sacrament of Confession, and the Sacrament of Holy Communication [53]. Nevertheless, the pandemic affected many religious practices due to the measures that were taken in order to prevent the spread of COVID-19 and to preserve public health. More specifically, entry to churches, monasteries, shrines as well as public worship, contact with other believers, and religious tourism was prohibited. All of these restrictions placed on religious ceremonies were devastating, as they used to establish social ties and a sense of belonging, a necessary component of wellbeing. Additionally, believers no longer had the opportunity to pray in religious places as they did before and could only pray at home [52]. All of these changes in the way that people expressed their religiousness are also the reason why spirituality did not positively predict the wellbeing components in this study. 

### 4.3. The Relationships between Human Strengths and the Wellbeing Component of Positive Relationships 

Under the particular quarantine conditions, positive relationships as a particular component of wellbeing were found to be positively predicted the highest by love and hope but also by social intelligence, gratitude, persistence, curiosity, and forgiveness. 

Positive relationships create feelings of belonging, security [26], and connection, which are the core factors of love. Connecting is about familiarity and caregiving [49]. It has been suggested that the higher levels of love that people demonstrate, the higher levels of the loving interactions with others someone will receive, and this relationship positively predicts positive relationships [27]. In addition, this character strength seems to play a key role in starting and maintaining relationships [27]. Loneliness has been positively associated with depressive and anxious symptoms while hope has been negatively linked to these symptoms. Hope can help people set goals, make thoughts about how to achieve them, and have less negative thoughts, which can be harmful [54]. In this way, individuals are able to create and maintain positive relationships, especially in difficult times such as quarantine, where it was very important for everyone to keep their hope levels high. 

As it can be seen from what was mentioned above, the wellbeing levels of people depend on the wellbeing levels of the people who they connect with [26]. In this way, some people have high levels of social intelligence, and this means that people with the ability to understand the feelings and motivations both of themselves and others [20] can maintain their positive relationships, which can lead them to higher levels of wellbeing. Gratitude gives individuals the chance to recognize every positive element of their relationships and increase the vigor of those relationships, as gratitude means being aware of and thankful for the good things that happen [20]. Concerning persistence, this character strength is about finishing what one starts and overcoming difficulties [20,48]. Assuming that quarantine is a situation characterized by social isolation [7,13], the strength of persistence seems to be crucial in maintaining positive relationships. Curiosity is about exploring, taking interest in experiences [20], and desire to acquire new knowledge. The positive relationship between curiosity and positive relationships in the present study might be social curiosity. This means the desire to explore and understand the motivations, behaviors, and feelings of others [55]. In a new situation such as quarantine, it was important to understand how others perceived what was happening, how others spent their time, and how they coped with the related difficulties. Social curiosity, conceivably, brought people closer, even under these conditions. Forgiveness means to forgive the wrong that other people have done [20], and social curiously positively predicted positive relationships, perhaps via the way that some people found quarantine to be an opportunity to care about their personal relationships [45]. 

On the other hand, negative correlations were found with the appreciation of beauty and spirituality. Appreciation of beauty has not ever been strongly correlated to positive relationships [22,27]. Maybe this negative relationship can be explained by the fact that this character strength is about noticing excellent performance, and during quarantine, due to the isolation [7,13], it was difficult to recognize both the performance of others and the beauty of their close and positive relationships, so this strength could not lead to positive relationships [7,13]. Regarding spirituality, an explanation was given above.

### 4.4. The Relationships between Human Strengths and the Meaning in Life Component of Wellbeing

Again, under these particular conditions of quarantine, meaning in life, as a particular component of wellbeing, was found to be positively predicted the highest by appreciation of beauty, leadership, and zest.

Meaning in life creates motivation and passion for people with respect to their lives but also fulfills them and correlates to the experience of positive emotions. Another relation between meaning and positive emotions is the procedure of anticipating future pleasant occurrences, appreciating current positive events, or reminiscing about past pleasant occurrences. An individual’s sense of meaning during positive events in their lives is enhanced by these strategies. As one’s life is enhanced, more good emotions are felt, and psychological flourishing can take place [26].

Appreciation of beauty is related to observing and appreciating beauty, excellence, and gifted execution in all spaces of life [20]. It was an important source of meaning in people’s lives [27] because taking note and appreciating the positive aspects of life, despite the crisis around [56], can lead people to higher levels of meaning in their life and consequently to higher levels of wellbeing. 

Leadership is about organizing and ensuring the success of group activities [20]. This positive relationship between leadership and meaning in life can be explained by the fact that in quarantine, people were isolated in their homes apart from every activity, hobby, work they had before [7,13]. Therefore, even the small goals and activities that were managed to be implemented during this period gave them a sense of meaning and fulfillment. 

Zest is about approaching life with fervor and vitality [20] and is one of the highest strengths that predicted wellbeing during quarantine. People with zest are searching for the meaning of life and reasons to live and are trying to create their future in the best way possible [57], no matter what the difficulties are. 

The findings showed negative correlations between hope, love, forgiveness, and meaning in life. According to the literature, hope is usually positively correlated to meaning in life. Regarding love and forgiveness, no significant positive correlation to meaning in life was found nor was a negative correlation [22,27]. At this point it should be mentioned that according to the *positive activity model* [58], there are some conditions under which character strengths and their followed positive activities can lead to happiness and wellbeing. Particularly, to achieve wellbeing, people need to perform positive activities. These positive activities increase the levels of wellbeing through some mediators, namely positive emotions, positive thoughts, positive behaviors, and need satisfaction. However, performing positive activities has some features. First, there are the features of the activity: *dosage:* the more the better, but it depends on every activity and every person; sometimes it is easy to exaggerate and have negative consequences. *Variety:* the literature supports that completing two to four positive activities concurrently may lead to higher levels of wellbeing. *Sequence:* this concerns which positive activity is the starter activity and *social support*. Second, there are the features of the person. To reach higher levels of wellbeing, people need to perform positive activities based on their character strengths with effort and high motivation. In the end, person–activity fit, which is how much the features of the activity are close to the features of every personality, further predict the levels of wellbeing.

In this study, because people were in quarantine conditions, not all of the features could be utilized, so even though people still generally had and used their specific character strengths, they could not apply them by performing positive activities in order to achieve higher levels of wellbeing due to the specific conditions. People in quarantine are isolated in their homes, are unable to engage in social interactions, and are faced with a lot of restrictions, so they have fewer opportunities to perform all of the positive activities that they used to [7,13]; for example, there are limited opportunities use humor by making jokes or love by expressing their feeling or by spending time with family and friends.

### 4.5. The Relationships between Human Strengths and Wellbeing as Accomplishment

As a specific component of wellbeing, under these specific quarantine conditions, accomplishment was found to be positively predicted the highest by persistence but also by perspective, zest, love, leadership, curiosity, hope, love of learning, and self-regulation. 

Accomplishment motivates people to set goals and achieve them. This process is achieved through the desire to succeed. The best possible performance is rewarded both by oneself and by society. Of course, judging one’s success is completely subjective. Subsequently, accomplishment can increase wellbeing and reduce anxiety and depression levels. An important factor in the accomplishment process is social support, as it helps people set and achieve more difficult goals [26].

It has been found [27] that accomplishment can be the most strongly and most consistently predicted by perspective, persistence, and zest. In accordance with what was mentioned above about people and accomplishment in quarantine, perspective is required to arrange and set appropriate long-term goals, while persistence and zest are vital to arrange and pursue goal pursuit and to encourage objective fulfillment [27]. 

In this way, love can play an important role, as it contributes to the value of close interpersonal relationships [20]. During the quarantine, people had the opportunity to strengthen their relationships with their loved ones either by living in the same house or by communicating through technology, which helped more than ever. At the same time, during the quarantine, many people either continued to work in the same or in a different way than they did before or found new job opportunities and set new goals [45]. In all these circumstances, social support, which is often achieved through love, played an important role. Ιn the process of starting new activities and succeeding, leadership seemed to help because this is the purpose of leadership [20], and leadership seemed to positively predict accomplishment; likewise, curiosity makes ongoing experiences more interesting [20], and this character strength might help people in quarantine to find their new—or in a new way—activities that are more interesting, and for this reason this was achieved. As long as accomplishment motivates people to set and achieve goals, during the quarantine period, hope allows people to anticipate the best andgives them hope that they will achieve it while at the same, time love of learning creates the tendency to perfect new skills and acquire new knowledge in people [20]; indeed, many people found quarantine to be a period to achieve these goals [45]. Self-regulation is about regulating feelings and behaviors [20]; therefore, it is useful for humans to reduce stress levels so as not to negatively affect them in order to achieve the goals they set.

However, a negative correlation with spirituality has been found. Again, spirituality can promote mental health through the power of faith [42,51]. Religiousness, as an integral and crucial component of spirituality, provides believers support, confidence, and hope [59]. Additionally, spirituality is an important source of support for dealing with difficulties [59]. In order to connect with God and feel supported, many people need to visit churches, shrines, come in contact with other believers, and show public worship. However, all of these practices, which are a source of strength for believers, were prohibited during quarantine, as people were isolated in order to ensure public health [52].

### 4.6. The Relationships between Virtues and Subjective Wellbeing Components

It was found that interpersonal virtue positively predicted state negative affect and negatively predicted state positive affect. This finding is in contrast with the literature [22,46], where the opposite relationship seems to be found. It has been proposed that quarantine was a period where interpersonal relationships were tested, people had to find new ways to communicate and adapt to them, and was also a period that caused intense anxiety about the health of the people close to them [45].

Maybe these findings can be explained by the fact that the period that was studied in the present study was the first days of the fist quarantine, where people were isolated in this way for the first time and may have not yet adapted to the new situation. Additionally, another explanation could be given by the “broaden and build theory”, which was described above [44]. Additionally, it was found that interpersonal virtue negatively predicted accomplishment. Accomplishment includes effort and success, and social support plays an important role [21,26], which, in the specific period studied here, could not exist, as it was needed for as was also the case for social relationships, as described above, because of the isolation. However, interpersonal virtue positively predicted positive relationships and meaning in life. Although quarantine was a period where interpersonal relationships were tested, some relationships became stronger, and people found new ways to communicate [45]. Consequently, the distance and the isolation may have created negative emotions due to the lack of face-to-face interaction, but close relationships were tested and became stronger and more meaningful.

Intellectual virtue was found to positively predict state positive affect, positive emotions, engagement, and accomplishment. These relationships might have occurred due to the strong prerequisites of having to adapt to a new way of life. Intellectual virtue seems to help people have a higher ability to adjust to the requests of the environment. During this lockdown period, the population was required to learn diverse ways of working, studying, communicating, and exercising, and the strengths of this virtue played a key role for people to cope with the new reality [46].

Virtue of restraint positively predicted accomplishment. Some people found the quarantine period to be a chance to set goals or to try to achieve goals they already had [45]. However, quarantine created very stressful conditions in which people needed to adapt to a new reality, creating a new way of achieving their goals. This process was too difficult, especially for those with low in restraint strengths because the situation demanded self-control. As such, people who are high in restraint strengths were more easily able to cope with the stress and difficulties, have self-efficacy, and finally, were able to achieve their goals [46]. 

Virtue of transcendence was found to positively predict state positive affect, positive emotions, engagement, positive relationships, and accomplishment. It was also found to negatively predict state negative affect and meaning in life. This virtue increases positive emotions and reduces negative emotions, through the power of faith, and in this way, promotes wellbeing [50,51]. Additionally, it enhances engagement because it motivates people for the future and makes them more capable of coping with difficulties, such as quarantine and the consequent security measures, and accomplish their goals [46,47]. Nevertheless, due to quarantine and the isolation, people were restricted and not able to do the activities that they did before [46], such as going to the church, so this may be the reason why the transcendent virtue negatively predicted meaning in life. 

### 4.7. The Relationship between Individual Demographic Characteristics, Virtues and Subjective Wellbeing

In the present study, demographic characteristics, except age, did not find to correlate with any virtue or with any wellbeing component. It is worth mentioning that during quarantine, many people were suspended or downsized from their jobs. Although this variable was not examined in terms of in terms of how it moderated the relationships that were studying (character strengths and wellbeing), we assume that this fact would have certainly affected the mental health indicators.

Age positively predicted all virtues apart from the intellect virtue, where there no correlations were found. These findings are in line with the literature, according to which, in the age- group of people aged 45 plus, the virtues of transcendence, restraint, knowledge, and the interpersonal virtue were found to reach higher levels compared to younger adults [22]. 

However, in the present study, age was shown to negatively predict positive emotions and positive relationships. According to the literature, it has been proven that older adults have more positive emotions compared to younger adults [60,61,62,63]. Older adults use more effective emotion-regulation strategies, and they are oriented to experience more positive emotions. They are aware of the limited future, so they are focused on having positive experiences and positive emotions, which is in contrast with younger adults, who are oriented to acquire knowledge [60,62]. Nevertheless, in extremely negative and changeable situations, older adults are more vulnerable and present more consequences and difficulties in coping with them [62,64].

Generally speaking, the sample in this study mainly consisted of younger and middle-aged adults, so the negative effect of age on positive emotions and positive relationships may reflect the lower levels of positive emotions and relationships in these populations due to the lack of older adults.

## 5. Conclusions

The present research examined whether associations between character strengths can promote wellbeing in adverse situations, such as quarantine. The findings showed that the character strengths that predict subjective wellbeing in quarantine the most are love, curiosity, persistence, hope, and zest. Additionally, it was found that under the specific condition of quarantine, most of 24 human strengths are “able to act” to maintain or enhance subjective wellbeing. This finding is very important in terms of developing easy and effective interventions in order for adults to cope with this adverse situation. However, some character strengths, especially spirituality, have a negative correlation with specific wellbeing components during quarantine. In any case, this “differentiation” is not able to neutralize the general positive role of human strengths in wellbeing enhancement. Age was the only demographic that moderated this relationship by positively predicting all of the virtues except intellectual virtue and negatively predicted two components of wellbeing, namely positive emotions and positive relationships.

## 6. Theoretical and Practical Implications

The contribution of the present research is of great importance both in the study of character strengths and virtues under difficult and unexpected situations such as quarantine as well as in the promotion of mental health during these periods. The results of the research, in addition to their contribution to the field of positive psychology, can be used by counsellors, coaches, and psychologists in education, work, or clinical settings in order to design and implement interventions that assist individuals in identifying the “good in their core”, their character strengths, cultivate them, and apply them in their everyday lives in order to achieve higher levels of wellbeing and its components. Last but not least, the results of the present study are of high importance for social and health policy not only in Greece but for other countries as well since public health and work organizations could apply interventions to enhance the implementation of character strengths in people’s lives, creating more resilient people through the COVID-19 pandemic as well as also happier citizens and happier societies.

## 7. Limitations and Recommendations for Future Research

The present study, despite the significant findings, had some limitations. The sample was convenient. Furthermore, the sample mainly consisted of women, which may be due to the fact that the population of Greece mainly consists women and because women were probably more receptive to participate voluntarily in the research, something that is often observed in research in the Greek population. In addition, the mean age of the sample was 32.08 years of age. The results were based on self-report questionnaires; thus, response bias could have taken place. The whole procedure was completed online, which means that some participants may have made some mistakes due to a lack of familiarity or haste. Additionally, the researchers studied the whole spectrum of 24 character strengths and 5 virtues of positive psychology, so it was very difficult the exact causal relationships for every character strength that was studied.

For future research, we propose that the relationships between each of the 24 character strengths be studied in more detail with respect to wellbeing via a longitudinal design, a wider age range, and a sample that is more representative of the Greek population under psycho-stressful and unprecedented conditions such as the pandemic and the subsequent quarantine to determine the exact relationship between virtues and character strengths with subjective wellbeing components in such situations. 

## Figures and Tables

**Table 1:** The five virtues and their character strength constituents.

Virtues	Character Strengths
Interpersonal Virtue	Forgiveness, Modesty, Appreciation of beauty, Kindness, Love, Teamwork, Fairness, Leadership
Virtue of Intellect	Curiosity, Open-mindedness, Creativity, Perspective, Bravery, Social intelligence, Humor
Virtue of Restraint	Self-regulation, Prudence, Persistence, Honesty
Transcendence Virtue	Gratitude, Hope, Spirituality, Zest
Virtue of Knowledge	Love of Learning

**Table 2:** Correlations between interpersonal virtue dimensions and subjective wellbeing components.

	Negative Affect State	Positive Affect State	P	E	R	M	A	Forgiveness	Modesty	Appreciation of Beauty	Kindness	Love	Teamwork	Fairness	Leadership
**Negative Affect State**															
**Positive Affect State**															
**P**	−0.175														
**E**		0.170	0.494												
**R**			0.404	0.280											
**M**	0.395		−0.182												
**A**	−0.176	0.154	0.539	0.370	0.177										
**Forgiveness**															
**Modesty**								0.352							
**Appreciation of Beauty**								0.277	0.218						
**Kindness**								0.400	0.355	0.319					
**Love**								0.282	0.227	0.273	0.399				
**Teamwork**								0.432	0.463	0.305	0.571	0.346			
**Fairness**								0.534	0.359	0.255	0.573	0.303	0.630		
**Leadership**								0.350	0.408	0.296	0.474	0.282	0.624	0.531	

**Table 3:** Correlations between virtue of intellect dimensions and subjective wellbeing components.

	Positive Affect State	Negative AffectState	Positive Emotions	Engagement	Positive Relationships	Meaning in Life	Accomplish-ment	Curiosity	Open Mindedness	Creativity	Perspective	Bravery	Social Intelligence	Humor
**Positive Affect State**														
**Negative Affect State**														
**Positive Emotions**		−0.269				−0.243								
**Engagement**		−0.109	0.489											
**Positive Relationships**		−0.151	0.546	0.288		−0.121								
**Meaning in Life**		0.429												
**Accomplishment**		−0.238	0.508	0.334	0.201									
**Curiosity**														
**Open Mindedness**								0.324						
**Creativity**								0.721	0.392					
**Perspective**								0.333	0.552	0.455				
**Bravery**								0.240	0.325	0.378	0.407			
**Social Intelligence**								0.434	0.381	0.450	0.454	0.280		
**Humor**								0.474	0.205	0.381	0.266	0.185	0.418	

**Table 4:** Correlations between virtue of restraint dimensions and subjective wellbeing components.

	Positive Affect State	Negative Affect State	Positive Emotions	Engagement	Positive Relationships	Meaning in Life	Accomplishment	Self Regulation	Prudence	Persistence	Honesty
**Positive Affect State**											
**Negative Affect State**											
**Positive Emotions**	0.290	−0.255				−0.175					
**Engagement**	0.336	−0.115	0.599								
**Positive Relationships**	0.132	−0.119	0.595	0.384							
**Meaning in Life**		0.409									
**Accomplishment**	0.268	−0.259	0.586	0.447	0.310						
**Self Regulation**											
**Prudence**								0.401			
**Persistence**								0.359	0.408		
**Honesty**								0.287	0.448	0.509	

**Table 5:** Correlations between transcendent virtue dimensions and subjective wellbeing components.

	Positive Affect State	Negative Affect State	Positive Emotions	Engagement	Positive Relationships	Meaning in Life	Accomplishment	Gratitude	Hope	Spirituality	Zest
**Positive Affect State**											
**Negative Affect State**											
**Positive Emotions**		−0.121									
**Engagement**	0.171		0.479								
**Positive Relationships**			0.470	0.266							
**Meaning in Life**	0.091	0.353	−0.167								
**Accomplishment**	0.103	−0.164	0.430	0.317	0.137						
**Gratitude**											
**Hope**								0.622			
**Spirituality**								0.637	0.527		
**Zest**								0.621	0.747	0.551	

**Table 6:** Correlations between virtue of knowledge dimensions and subjective wellbeing components.

	Positive Affect State	Negative Affect State	Positive Emotions	Engagement	Positive Relationships	Meaning in Life	Accomplishment	Love of Learning
**Positive Affect State**					0.147			
**Negative Affect State**								
**Positive Emotions**	0.307	−0.265			0.621	−0.213		
**Engagement**	0.322	−0.126	0.606		0.397			
**Positive Relationships**		−0.173						
**Meaning in Life**		0.414			−0.125			
**Accomplishment**	0.294	−0.236	0.619	0.469	0.337			
**Love of Learning**								

**Table 7:** Correlations between demographics, virtues, and subjective wellbeing components.

		**Positive Affect State**	**Negative Affect State**		**Positive Emotions**	**Engagement**	**Positive Relationships**	**Meaning in Life**	**Accomplishment**	**Interpersonal Virtue**	**Virtue of Intellect**	**Virtue of Restraint**	**Transcendent Virtue**	**Virtue of Knowledge**
**Positive Affect State**														
**Negative Affect State**														
**Positive Emotions**			−0.217											
**Engagement**		0.134			0.491									
**Positive Relationships**			−0.195		0.533	0.218								
**Meaning in Life**			0.409		−0.212		−0.160							
**Accomplishment**		0.110	−0.217		0.513	0.358	0.250							
**Interpersonal Virtue**											0.524			0.181
**Virtue of Intellect**														
**Virtue of Restraint**										0.475	0.381			0.145
**Transcendent Virtue**										0.613	0.515	0.479		0.221
**Virtue of Knowledge**											0.359			

## Data Availability

The data presented in this study are available on request from the corresponding author.
